# Increasing bowel cancer screening using SMS in general practice: the SMARTscreen cluster randomised trial

**DOI:** 10.3399/BJGP.2023.0230

**Published:** 2024-03-19

**Authors:** Jennifer G McIntosh, Mark Jenkins, Anna Wood, Patty Chondros, Tina Campbell, Edweana Wenkart, Clare O’Reilly, Ian Dixon, Julie Toner, Javiera Martinez-Gutierrez, Linda Govan, Jon D Emery

**Affiliations:** Centre for Epidemiology and Biostatistics, University of Melbourne, Melbourne.; Centre for Epidemiology and Biostatistics, University of Melbourne, Melbourne.; Department of General Practice and Primary Care, University of Melbourne, Melbourne.; Department of General Practice and Primary Care, University of Melbourne, Melbourne.; Healthily Pty Ltd, Melbourne.; Pen Computer Systems, Sydney.; Workforce Development, Screening Early Detection and Immunisation, Cancer Council Victoria, Melbourne, and executive manager, Chronic Health, Population Health Unit, VACCHO, Melbourne.; Melbourne.; Melbourne.; Department of General Practice and Primary Care, University of Melbourne, Melbourne.; Ballarat Goldfields/Wimmera Grampians, Western Victoria Primary Health Network, Ballarat.; Department of General Practice and Primary Care, University of Melbourne, Melbourne.

**Keywords:** bowel cancer screening, colorectal cancer, general practice, primary health care

## Abstract

**Background:**

Australia has one of the highest incidences of colorectal cancer (CRC) worldwide. The Australian National Bowel Cancer Screening Program (NBCSP) is a best-practice, organised screening programme, but uptake is low (40.9%) and increasing participation could reduce morbidity and mortality associated with CRC. Endorsement by GPs is strongly associated with increasing screening uptake.

**Aim:**

This study (SMARTscreen) aimed to test whether a multi-intervention short message service (SMS) sent by general practices to 50–60-year-old patients who were due to receive the NBCSP kit would increase NBCSP uptake, by comparing it with usual care.

**Design and setting:**

A stratified cluster randomised controlled trial was undertaken, involving 21 Australian general practices in Western Victoria, Australia.

**Method:**

For intervention practices, people due to receive the NBCSP kit within a 6-month study period were sent an SMS just before receiving the kit. The SMS included a personalised message from the person’s general practice endorsing the kit, a motivational narrative video, an instructional video, and a link to more information. Control practices continued with usual care, comprising at-home testing with a faecal immunochemical test (FIT) through the NBCSP. The primary outcome was the between-arm percentage difference in uptake of FIT screening within 12 months from randomisation, which was estimated using generalised linear model regression.

**Results:**

In total, 39.2% (1143/2914) of people in 11 intervention practices and 23.0% (583/2537) of people in 10 control practices had a FIT result in their electronic health records — a difference of 16.5% (95% confidence interval = 2.02 to 30.9).

**Conclusion:**

The SMS intervention increased NBCSP kit return in 50–60-year-old patients in general practice. This finding informed a larger trial — SMARTERscreen — to test this intervention in a broader Australian population.

## Introduction

Colorectal cancer (CRC) in Australia contributes to a notable burden of disease that remains consistently high, with approximately 15 000 new diagnoses of CRC and 5000 CRC-related deaths per year (11% of all cancer deaths).^1^ Only two-thirds of people diagnosed with CRC are expected to live beyond 5 years after diagnosis,^1^ and treatment costs the Australian Government A$1.1 billion (£571 million) per year.^2^ This high burden of disease is despite CRC being considered a cancer that can be both prevented (through the detection and removal of pre-cancerous polyps) and detected early on (stage-one CRC has a 99% relative survival rate).^1^

The Australian National Bowel Cancer Screening Program (NBCSP) is an Australian Government initiative aimed at reducing morbidity and mortality from CRC by offering a free, non-invasive screening test to detect early signs of CRC.^3^ Established in 2006, and expanded in subsequent years, it involves all Australians aged 50–74 years being mailed a self-collection faecal immunochemical test (FIT) kit every 2 years. Although this is a simple and cost-effective screening method for CRC,^4^ completion and returning of kits remains low at 40.9%.^5^ Participation is lowest in the 50–60-year-old age groups, with only 33.4% returning their kits.^5^ It has been estimated that increasing uptake of the NBCSP from 40% to 60% would prevent an additional 37 300 diagnoses of CRC and 24 800 deaths from CRC, as well as reducing the annual health budget expenditure by A$2.1 billion by 2040.^4^

Effective interventions to increase screening uptake have included endorsement of CRC screening by a GP,^6^^–^8 TV public-service announcement campaigns featuring relatable people discussing their personal screening experience,^9^ and the provision of practical, easy-to-follow instructions about completing the NBCSP kit.^10^^,^^11^ Short message service (SMS) messaging has been demonstrated to have an impact on screening uptake for first-time screeners in the UK,^12^ as well as in Alaska Natives and people of American Indian ethnicity.^13^ A systematic review by Uy *et al* demonstrated that SMS reminders increased breast and cervical screening.^14^ Each of these different interventions (that is, GP-endorsement, positive reinforcement of screening by relatable people, and clear instructions) have been tested individually, but not in combination. Evidence supports the use of combining effective health-promotion interventions to increase the uptake of screening,^15^^,^^16^ which was the justification for the SMARTscreen trial.

The trial examined whether general practice patients aged 50–60 years who were due for screening and received the SMARTscreen intervention were more likely to complete CRC screening than similar general practice patients in the control arm. The secondary aim, not reported here, was to evaluate whether sending the SMS was acceptable and feasible to general practice staff and their patients.

**Table:** How this fits in

Australia has a persistently high rate of colorectal cancer and a low rate of screening. Every 2 years, the Australian National Bowel Cancer Screening Program (NBCSP) sends a faecal occult blood test to every Australian aged 50–74 years old. Increasing screening from the current rate of 40.9% has the potential to greatly reduce mortality and morbidity. Endorsement of screening by general practice is considered one of the most effective ways to increase participation in cancer screening programmes. The SMARTscreen trial tested the efficacy of general practices sending patients an endorsement SMS message, along with a combination of web-based motivational and instructional videos, just before they received their faecal immunochemical test kit. The proportion of patients who participated in the NBCSP was 16.5% higher in the intervention arm than the control arm, over 12 months. This finding is important, as the intervention has the potential to be implemented into clinical practice to increase uptake of screening throughout Australia.

## Method

A detailed trial protocol was published before practices were recruited, and the trial was conducted with no substantive changes to the protocol.^17^ CONSORT guidelines^18^ were used for reporting the trial.

### Trial design

This was a stratified cluster randomised controlled superiority trial. General practices were randomly allocated, in a 1:1 ratio, to two trial arms, stratified by practice location and practice size according to the number of patients. The unit of randomisation was at practice level for practical reasons, including the technical implementation of the intervention, the fact that the outcome could only be collected in aggregate form from the practice’s electronic health records (EHRs), and to minimise the risk of contamination; for example, if two family members from the same practice were randomised to different arms of the trial.

### Setting

General practices were located in the Western Victorian Primary Health Network (WVPHN) geographical catchment area in regional Victoria, Australia.^19^ This region comprises a mixture of cities and towns, with a growing population of older, and therefore eligible, people, and >200 general practices.^19^

### Participants

#### Eligibility criteria

General practices were eligible if they were:

- from the WVPHN region (which covers the geographical location classified as Modified Monash Model [MMM] 1 to 5);^20^ and
- had at least two full-time equivalent GPs.

People from eligible practices were included if they were ‘active patients’; that is, if they:

- had had at least three medical consultations at the practice within the previous 2 years (as defined by the Royal Australian College of General Practitioners);^21^
- were aged 50–60 years old (inclusive); and
- were due to receive the NBCSP kit in the 6-month intervention period. The due date for the NBCSP kit was determined either by birthday (NBCSP kits are sent every 2 years from a patient’s 50th birthday until they are 74 years old), or 2 years after a previous NBCSP kit had been received; if a person completes a kit, the date the next kit is due will be 2 years after the date of completion.

Individuals were excluded if they:

- did not have a recorded mobile phone number in the EHR;
- had a diagnosis of CRC in their medical records; or
- had opted out of receiving SMS messages from their practice.

### Intervention

SMARTscreen aimed to test the effect on screening participation of a combination of four evidence-based interventions delivered in an SMS from a general practice to their patients, by comparing it with usual care. Usual care included screening with the NBCSP. The trial was restricted to 50–60-year-olds, as they have a high level of daily smartphone use^22^ and that age group had the lowest screening rates.^5^

The SMS intervention included:

- an endorsement of the NBCSP by the person’s own general practice;
- a video of a person telling a positive story about how completing the kit affected them;
- an animated instructional video demonstrating how to complete the kit; and
- a link to information about CRC, the NBCSP and the importance of screening.

The SMARTscreen intervention was co-designed by the investigator team, which included clinical, academic, consumer, and industry representatives. Using existing evidence-based resources, along with an iterative process, the investigator team developed an SMS that included a text message with an embedded weblink, which opened to a customised webpage with four screens designed to increase screening.^17^ Each weblink had a unique alphanumeric code to enable tracking of the number of times each webpage was opened. An SMS was sent from the person’s general practice to each eligible individual in the last week of the month before their kit was due. Details on how patients were identified are outlined later in this article.

The trial intervention period started on 1 January 2021 and ended on 30 June 2021. The outcome assessment ran over a 12-month timeframe, from 1 January 2021 until 31 December 2021. Some practices had technical difficulties installing the software required for the trial, which delayed their start date by 1 month; as such, their intervention period started on 1 February 2021 and ended on 31 July 2021, and their follow-up period ran from 1 February 2021 until 31 January 2022.

In the first 3 months of the intervention period, the videos in the SMS weblink needed to be actively clicked to start playing; after 3 months, the technology changed and the video content automatically played when the weblink was opened. As such, after the first 3 months, it was no longer possible for the authors to measure whether people intentionally opened the video to watch it.

### Outcome

The primary outcome was the difference, between the intervention and control arms, in the proportion of eligible people who completed a FIT for up to 12 months after the SMS was sent, as recorded in the EHR.

### Data collection

The proportion of eligible people who completed a FIT was calculated by dividing the total number of eligible people who had an NBCSP FIT kit result recorded in the EHR at participating practices (numerator) by the total number of individuals eligible to receive a FIT from the NBCSP at each practice during the study period (denominator). Individuals eligible to receive a FIT in the next 6 months (denominator) were extracted from general practice EHRs using the CAT4 clinical audit tool, along with specific filters that were developed to determine when the patients’ kits were due, based on either their birthday or 2 years after their previous kit was returned. Individual-level data were stored at the general practice and each SMS was sent by the practice directly to the eligible people. Using a data-collection template, the trial coordinator at each practice recorded the number of individuals who were eligible to receive a FIT kit by the month the SMS would be due, at ages 50, 52, 54, 56, 58, 60 years, or as undetermined age, and sex (male/female). No identifying information was extracted.

To determine the number of individuals with an NBCSP FIT kit result recorded in the EHR, an audit of the EHRs in each general practice was conducted by the trial coordinator. Information was collected on the patient’s month and year of birth, sex (male/female), and date of the FIT result. The date for the recorded FIT result was defined as the date the general practice received a copy of it in the EHR from the NBCSP, and had to fall between 1 January 2021 and 31 December 2021, or between 1 February 2021 and 31 January 2022 for practices with a delayed start. Within this dataset, the authors also determined the month that individuals were due to receive the SMS, based on the month of their birthday.

For statistical analysis of the primary outcome, the two data sources containing the number of individuals eligible for the trial (denominator) and number of eligible individuals that had a recorded FIT result in the EHR (numerator) were counted for each general practice and merged at the aggregate level by general practice. The geographical location of the general practice was classified using the MMM, and the number of active patients aged 50–60 years was collected for each general practice.

### Sample size

For 80% power with a two-sided 5% alpha level, the authors required 1400 eligible general practice patients from 20 practices (70 patients per practice) to detect a 10% increase in proportion with recorded bowel cancer screening in the intervention arm (50%) compared with the control arm (40%), assuming an intra-cluster correlation coefficient of 0.008.^23^

### Randomisation

#### Sequence generation

General practices were randomised at a ratio of 1:1 into intervention or control arms. The allocation sequence was computer generated by a statistician, stratified by geographical remoteness (MMM 1–3 and 4–5) and general practice size (<1000 active patients versus ≥1000 active patients), using random permuted block sizes of two and four in each stratum.

#### Allocation concealment

The block sizes were not disclosed until data collection and primary analysis had been completed. Before randomising practices, the trial coordinator randomly assigned the codes 1 and 2 to the intervention and control arms, and created a masked key, which was kept separate from the random-allocation schedule. After practices consented to the trial and baseline data were collected, the statistician randomly assigned the practice to arm 1 or 2 using the random-allocation schedule. The study coordinator was then notified, and it was then that they unmasked the allocation and informed the practice to which trial arm they had been allocated.

#### Implementation

The trial coordinator met with the general practice staff via Zoom or in person, after which a delegate for the practice provided written consent to participate on behalf of the practice. All staff were given written information about the trial and the methods before consenting. Consent was only given if all GPs in the practice agreed to be involved.

#### Blinding

Practices and the trial coordinator could not be blinded for pragmatic reasons. The masked key for the trial arm allocation was only revealed to the statistician and those investigators not involved in the trial implementation after the blinded review of the primary analysis.

### Statistical methods

The characteristics of each general practice and their eligible patients were summarised using counts and percentages by trial arm. Primary analysis was intention to treat;^24^ this included all general practices and eligible individuals, irrespective of whether they received all, or part of, the intended intervention. For the primary analysis, individuals were excluded if they were outside of the age range, or for whom a FIT result was recorded in January 2021 if their general practice started the intervention in February 2021.

Generalised linear models, using the logit and identity link function, with binomial family, adjusted for the randomisation stratification factors (geographical remoteness and general practice size) were used to estimate the odds ratio and difference in proportions for the primary outcome between the two trial arms, respectively. Generalised estimating equations with robust standard errors were used with the models to allow for clustering by general practice. The estimated intervention effect was reported as the odds ratio and the percentage difference between trial arms with respective 95% confidence intervals (CIs). *P*-values were estimated using the logistic regression model described above. The intra-practice correlation coefficient was estimated using one-way analysis of variance and reported with 95% CI. Statistical analysis was conducted using Stata (version 17).

After a blinded review of the data, before any statistical analysis, three additional sensitivity analyses, using the same statistical methods as above, were included using different criteria for defining the numerator of the primary outcome:

- sensitivity analysis 1 — all individuals identified with a FIT result in the EHR, including people who did not meet the eligibility criteria (that is, those outside of the age range or with FIT results recorded in the EHR before the start of the trial period);
- sensitivity analysis 2 — only those individuals with an identified FIT result in the EHR, whose birthday was within the 6-month intervention period;
- sensitivity analysis 3 — individuals as defined in sensitivity analysis 2, who had returned their FIT kit within 190 days from when their SMS was due (defined as the end of the data-collection month).

### Trial registration

The trial was registered with the Australian New Zealand Clinical Trials Registry (ID: ACTRN12620001020976).

### Patient and public involvement (PPI) statement

Two members of the public who were self-described as cancer PPI representatives were directly involved in the design of the intervention and contributed to the trial methodology including supervision. They were active co-investigators and co-authors of this article.

## Results

### Participant flow and recruitment

**Figure 1.**
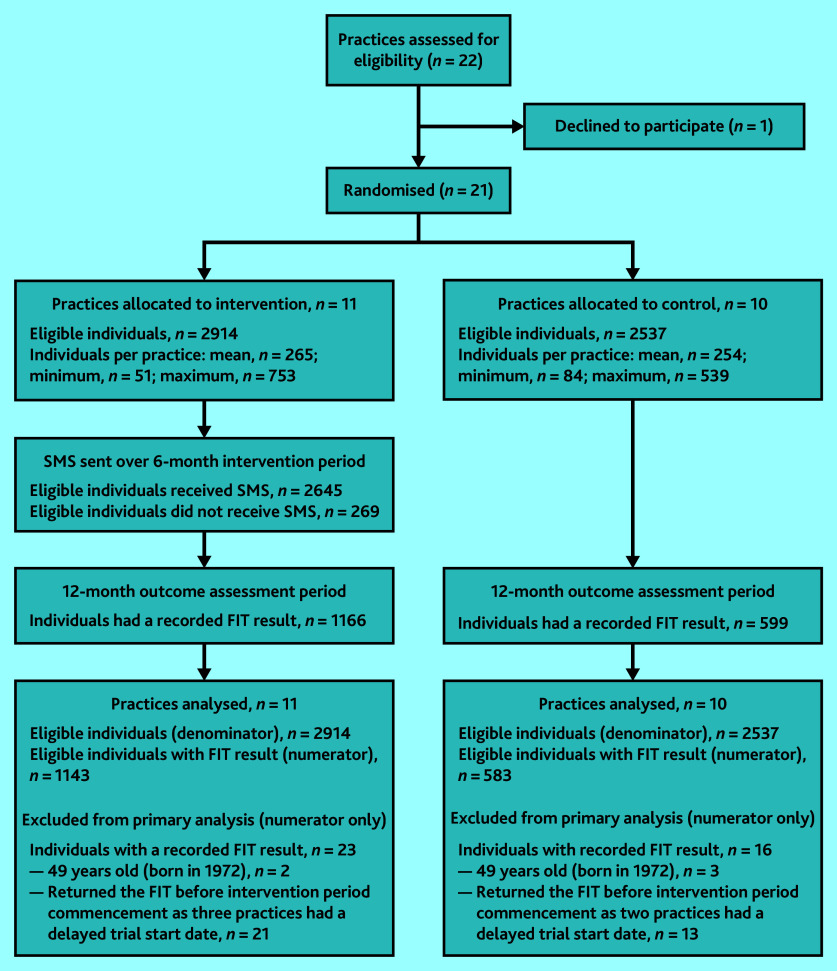
The SMARTscreen trial profile. FIT = faecal immunochemical test. SMS = short message service.

Figure 1 shows the trial profile. Between January 2021 and July 2021, 21 general practices of 22 that were approached were recruited into the trial; of these, 11 were allocated to the intervention arm and 10 were allocated to the control arm. Sixteen practices started the intervention period on 1 January 2021 as planned. Of the five practices that started their intervention period 1 month later, one was waiting for the CAT4 clinical audit tool to be installed, and the other four had technical issues (the clinical audit was not working and required repairs). In total, there were 5451 eligible individuals: 2914 (53.5%) in the intervention practices and 2537 (46.5%) in the control practices.

### Baseline data

Characteristics of general practices and eligible individuals were similar for the intervention and control arms (Tables 1 and 2).

**Table 1.** General practice characteristics

**General practice characteristics**	**Intervention, *n* (%)**	**Control, *n* (%)**
Practice trial-arm allocation	11 (52)^a^	10 (48)^a^

Geographical remoteness		
MMM 1–3	6 (55)	5 (50)
MMM 4–5	5 (45)	5 (50)

Practice size of patients aged 50–60 years		
<1000 patients	6 (55)	6 (60)
≥1000 patients	5 (45)	4 (40)

Date general practice started trial		
1 January 2021	8 (73)	8 (80)
1 February 2021^b^	3 (27)	2 (20)

a *Percentage of entire cohort (*n *= 21).*

b *Practices had a delayed start because of technical issues. MMM = Modified Monash Model for remoteness.*

**Table 2.** Individual characteristics (*n* = 5451 patients)

**Characteristics**	**Intervention**	**Control**
Total, *n* (%)	2914 (53.5)^a^	2537 (46.5)^a^
Mean number per practice, *n*	265	254
Range per practice, *n*	51–753	84–539

Sex, *n* (%)		
Female	1620 (55.6)	1389 (54.7)
Male	1294 (44.4)	1148 (45.3)

Biannual age when kit was due, *n* (%)		
50 years	474 (16.3)	419 (16.5)
52 years	348 (11.9)	347 (13.7)
54 years	364 (12.5)	335 (13.2)
56 years	332 (11.4)	360 (14.2)
58 years	323 (11.1)	337 (13.3)
60 years	368 (12.6)	295 (11.6)
Unknown to investigators^b^	705 (24.2)	444 (17.5)

NBCSP kit due date, *n* (%)		
Biennial birthday	2209 (75.8)	2093 (82.5)
2 years after previous kit returned	705 (24.2)	444 (17.5)

Date intervention period started,^c^ *n* (%)		
1 January 2021	2053 (70.5)	1756 (69.2)
1 February 2021	861 (29.5)	781 (30.8)

Month SMS was due (accounting for staggered start in the 6-month intervention period)		
1	433 (14.9)	395 (15.6)
2	468 (16.1)	394 (15.5)
3	504 (17.3)	430 (16.9)
4	516 (17.7)	412 (16.2)
5	489 (16.8)	453 (17.9)
6	504 (17.3)	453 (17.9)

a *Percentage of entire cohort of patients (*n *= 5451).*

b *Age of the individuals who were due to receive their NBCSP FIT kit 2 years after returning their previous one was not known to the investigators.*

c *Practices had a delayed start because of technical issues. NBCSP = National Bowel Cancer Screening Program. SMS = short message service.*

### Numbers analysed

The primary analysis included all 21 practices and all 5451 eligible general practice patients who were due to receive a FIT kit during the 6-month intervention period. This included 269 (9.2%) patients in the intervention arm, who did not receive the SMS.

In total, 1765 individuals (1166 in the intervention arm and 599 in the control arm) had a FIT result recorded in the EHR. Of these, 39 individuals were excluded from the numerator in the primary analysis as they did not meet eligibility criteria: five were born in 1972 and were just outside the eligible age range, and 34 had a FIT result recorded in their EHR in January 2021, but their practice started the intervention in February 2021.

### Outcomes and estimations

In total, 39.2% of individuals in the intervention arm had a FIT result recorded in the EHR compared with 23.0% of controls — an absolute increase of 16.5% (95% CI = 2.02 to 30.9; *P* = 0.03) in the 12-month follow-up period (Table 3). The estimated intervention effect was similar when all individuals identified with a FIT result were analysed (sensitivity analysis 1) (Table 3). Sensitivity analyses 2 and 3, where stricter definitions were applied to define the numerator (the denominator remained the same), showed that there was still a greater percentage of individuals who were due to receive a FIT kit in the intervention arm compared with the control arm.

**Table 3.** Primary outcome across 12 months for the intervention (*n* = 2914) and control (*n* = 2537) arms

**FIT result received**	**Intervention arm, *n* (%)**	**Control arm, *n* (%)**	**Difference in percentages,** %**^a^ (95% CI)**	**Odds ratio (95% CI)^b^**	***P*-value^b^**	**Intra-practice correlation coefficient (95% CI)**
**Primary analysis^c^**			16.5 (2.0 to 30.9)	2.3 (1.1 to 4.9)	0.03	0.148 (0.050 to 0.246)
**Yes**	1143 (39.2)	583 (23.0)				
**No**	1771 (60.8)	1954 (77.0)				

**Sensitivity analysis 1^d^**			16.5 (2.2 to 30.7)	2.3 (1.1 to 4.8)	0.03	0.145 (0.049 to 0.242)
**Yes**	1166 (40.0)	599 (23.6)				
**No**	1748 (60.0)	1938 (76.4)				

**Sensitivity analysis 2^e^**			10.5 (0.45 to 20.5)	2.1 (1.0 to 4.1)	0.04	0.084 (0.024 to 0.144)
**Yes**	706 (24.2)	369 (14.5)				
**No**	2208 (75.8)	2168 (85.5)				

**Sensitivity analysis 3^f^**			8.2 (−0.2 to 16.6)	1.9 (1.0 to 3.7)	0.06	0.066 (0.017 to 0.115)
**Yes**	594 (20.4)	318 (12.5)				
**No**	2320 (79.6)	2219 (87.5)				

a *Difference in percentages between arms and respective 95% CI estimated using generalised linear regression model (identity link function and binomial errors).*

b *Odds ratio, 95% CI, and* P*-value estimated using logistic regression. All regression models included randomisation stratification factors (geographical remoteness and general practice size) as fixed effects and used generalised estimating equations with robust standard errors to allow for a clustering effect of general practices.*

c *Includes all FITs received within 12 months from the start of the trial period for the practice (*n *= 1765), but excludes 39 people (five were born in 1972 and 34 had their FIT in January 2021, but their practices started the intervention in February 2021); final number of individuals with FIT results = 1726).*

d *Includes all people with a FIT test result who were identified (*n *= 1765), including the 39 people who were excluded because they were not eligible (number of individuals with FIT results = 1765).*

e *As for primary analysis (*n *= 1726), but excludes 651 individual FIT records for people whose birthday was outside of the 6-month intervention period (number of individuals with FIT results = 1075).*

f *As for sensitivity analysis 2, but only includes people whose FIT results were received between the end of the month the SMS was due and 190 days from the end of the month the SMS was due (number of individuals with FIT results = 912). FIT = faecal immunochemical test. SMS = short message service.*

### Harms

No harms or unintended consequences were reported for anyone involved in the trial.

## Discussion

### Summary

The SMARTscreen combination SMS resulted in the proportion of patients who participated in the NBCSP being 16.5% higher (95% CI = 2.02 to 30.9) in the intervention arm than the control arm, over 12 months. Given that it has been estimated that increasing screening participation by 10% could prevent 27 000 incident CRC diagnoses and 16 800 cancer deaths, and that an additional A$200 million expenditure could be saved over the next 20 years in the Australian population,^4^ these results provide compelling evidence for conducting a larger trial in the broader Australian population.

### Strengths and limitations

There were limitations to the data the authors were able to collect. Information about NBCSP results were extracted from the general practice EHRs, which may be incomplete.^25^ In addition, the percentage of eligible individuals who completed a FIT were likely to be underestimated, as only 80% of people aged 50–60 years old attend general practice every year,^26^ and not everyone nominates a GP to receive their NBCSP results. In the authors’ recent trial,^27^ 29.7% (131/441) of people did not have their NBSCP kit results in their patient record when compared with their NBCSP records; this was similar in both arms of the same trial (28.4% in the control arm, 30.8% in the intervention arm) (unpublished data). As such, the authors expect that the underestimation would be similar for both intervention and control practices, and the estimated absolute intervention effect would be unbiased. Nevertheless, the percentage of kits returned in the intervention arm was higher (39.2%) than the national average for 50–60-year-olds during the trial period (33.4% based on complete NBCSP data^5^).

The de-identified outcome data related to the total number of individuals eligible for the trial (denominator) and whether their FIT results were recorded in the EHR (numerator) were collected separately, so the authors were unable to link the individual-level data between the two data sources, but could link the data at the aggregate level for each practice. This limited the ability to conduct sub-group analyses to explore whether there were differences in the intervention effect by patient characteristics (for example, age groups).

Another limitation was not knowing whether people were receiving the SMS just before receiving their kit. The authors estimated when the kits would be received and timed the SMS for the month before this, according to the NBCSP rules. However, during the trial period, the NBCSP delivered kits up to 6 months after people’s birth dates, not always when they were due; this meant that it was possible that people did not receive the SMS just before they were due to receive the kit, but the authors had no way of assessing this.

The authors are addressing these limitations by using data from the newly established National Cancer Screening Register in a follow-on trial: SMARTERscreen.^28^ This trial is developing methods for collecting de-identified individual-level data directly from the register to ensure that the SMS is sent at the right time and the data collected are more complete; in this way, more-granular data can be provided about individual responses to the SMS, and it should be possible to ascertain the number of individuals with a FIT result, as recorded in the register.

Another potential limitation was the fact that the 12-month data collection period for each general practice was fixed from the time the practice entered the trial. However, the observation period for individuals varied, ranging from a minimum of 6 months to a maximum of 12 months, depending on the time between the SMS being sent and the end of the 12-month trial data-collection period. NBCSP monitoring data show that, if people are going to return their kit, most do so within 4 months;^25^ given that there was a minimum timeframe of 6 months in the study reported here, the authors were confident they would capture the bulk of returned kits. This was the same for both trial arms.

Unexpected problems were also encountered because the trial was conducted during the COVID-19 pandemic (2020–2021). However, co-design of the SMS was limited to existing evidence-based resources, expert opinion from the multidisciplinary investigator team, and consultation with PPI representatives. The authors were unable to conduct in-person consultations or focus groups with PPI as planned.

The recruitment of general practices was also challenging during the COVID-19 pandemic, with general practice facing unprecedented demands, including repeated lockdowns. Despite these challenges, the required number of practices were recruited with no attrition, and practice and patient characteristics between trial arms were similar.

The study was conducted in partnership with the WVPHN, and the sample was drawn from within the Western District of Victoria region. People living in that district live in, mostly, rural areas — half the practices were in areas categorised as MMM 4–5 (that is, either medium-sized or small rural towns) — and, as such, the findings might not be generalisable to the entire Australian population, the majority of whom live in metropolitan areas.^20^ As interventions have been demonstrated to be more effective in under-served groups,^29^ this result might not translate to other sub-populations with higher baseline screening rates. Also, the study was limited to the practices’ ‘active patients’, who were defined as having attended the practice at least three times within the previous 2 years.^21^ The authors excluded patients who were not regular attenders, as they assumed that ‘non-active’ patients would be less likely to nominate a GP in the trial when returning their NBCSP kit.

The results demonstrated that the SMARTscreen intervention led to the proportion of patients returning a kit being 16.5% higher; however, the screening participation data could be an underestimate for the reasons highlighted above, which would suggest the health and financial benefits might be even greater than suggested by the findings.

### Comparison with existing literature

These results demonstrate that developing a complex intervention that combines effective tools — namely, GP endorsement, narrative motivational videos, clear instructional videos, and information about bowel cancer screening — delivered via an SMS can have an effect on screening uptake that is equally as strong, or potentially greater, when packaged into one easily accessible intervention than using the individual components alone. The individual components found in previous research included a GP endorsement in a letter (11.8% increase),^7^ a mass-media campaign using positive narrative videos (15% increase),^9^ and an SMS alone (0.6% to 15%).^12^^–^14 The use of a multifaceted SMS intervention is supported by a systematic review of interventions to increase CRC screening uptake, which found that there was a compounded effect on screening when using a combination intervention instead of single interventions.^15^

### Implications for research

If expanded across the country, this intervention has the potential to result in large clinical and economic beneficial outcomes. SMARTscreen provided robust evidence to support a larger trial to test for the intervention’s effectiveness in the broader Australian community. The Australian National Health and Medical Research Council has funded a trial with a larger population as a direct result of the study reported here. The new trial, SMARTERscreen,^28^ will capture data using the new National Cancer Screening Register to provide complete datasets, along with patient characteristics such as age, sex, and location, to provide more individualised results. This will allow for a more-tailored or targeted approach to screening to be provided and, potentially, for there to be a focus on specific groups that currently under-screen. The results of SMARTERscreen will be available in 2025.

## Data

The data that support the findings of this study are available on request from the corresponding author. The data are not publicly available due to privacy or ethical restrictions.
